# Daily Marital Strain and Sleep in Same-Sex and Different-Sex Couples

**DOI:** 10.1093/geroni/igaa057.2045

**Published:** 2020-12-16

**Authors:** Michael Garcia

**Affiliations:** University of Texas at Austin, Austin, Texas, United States

## Abstract

Marital strain has consistently been linked to many indicators of daily health and well-being, including sleep. Prior studies show that, on days when marital strain is higher, women in different-sex couples experience poorer sleep outcomes. However, this work has not yet considered whether and how these relationships differ for men and women in same-sex couples. Using 10 days of dyadic diary data from 756 midlife U.S. men and women in 378 gay, lesbian, and heterosexual marriages, we examine the associations of daily marital strain with sleep quality and duration and consider whether these relationships differ across union type. Results suggest that increased marital strain is associated with poorer sleep quality and shorter sleep duration, but only for women married to men. These findings underscore the importance of including same-sex couples when exploring linkages between marital dynamics and health, especially when considering how gender impacts these processes.

